# Elevated expression of FABP3 and FABP4 cooperatively correlates with poor prognosis in non-small cell lung cancer (NSCLC)

**DOI:** 10.18632/oncotarget.10086

**Published:** 2016-06-15

**Authors:** Zhiyuan Tang, Qin Shen, Hao Xie, Xiaoyu Zhou, Jun Li, Jian Feng, Hua Liu, Wei Wang, Shu Zhang, Songshi Ni

**Affiliations:** ^1^ Department of Respiratory Medicine, Affiliated Hospital of Nantong University, Nantong 226001, Jiangsu, China; ^2^ Key Laboratory of Drug Metabolism and Pharmacokinetics, State Key Laboratory of Natural Medicines, China Pharmaceutical University, Nanjing 210009, Jiangsu, China; ^3^ Department of Pathology, Affiliated Hospital of Nantong University, Nantong 226001, Jiangsu, China

**Keywords:** fatty acid binding protein-3(FABP3), fatty acid binding protein-4(FABP4), non-small cell lung cancer, immunohistochemistry, prognosis

## Abstract

Fatty acid binding proteins (FABPs) are intracellular lipid-binding proteins that are involved in a variety of biological cellular processes, including tumorigenesis. In this study, we explored the expression pattern of FABP3 and FABP4 in non-small cell lung cancer (NSCLC) as well as their roles in prognosis. We determined mRNA expression of FABP3 and FABP4 in matched pairs of cancerous and non-cancerous fresh frozen tissues from 30 NSCLC patients. Tissue microarray immunohistochemical analysis (TMA-IHC) was applied to determine the protein expression of FABP3 and FABP4 in 281 cancerous and 121 matched adjacent non-cancerous tissue samples. Our results showed that both mRNA and protein expression of FABP3 and FABP4 were significantly higher in cancerous tissues when compared to non-cancerous tissues. Furthermore, high expression of FABP3 or FABP4 in NSCLC was significantly associated with advanced tumor node metastasis (TNM) stage and had a negative impact on the overall survival of NSCLC patients. Concurrent high expression of FABP3 and FABP4 was significantly related to TNM stage. In conclusion, our research demonstrated that high FABP3 or FABP4 expression had strong prognostic value for overall survival in NSCLC. Detection of FABP3 and FABP4 cooperatively was helpful to predict the prognosis of NSCLC.

## INTRODUCTION

Lung cancer is one of the primary causes of cancer-related death worldwide and greater than 80 percent of lung cancer patients have non-small cell lung cancer (NSCLC) [1-3]. Despite improvements in treatment modalities (such as surgical resection, chemotherapy, radiotherapy, and targeted therapy), the long-term survival of NSCLC is still dismal with 5-year survival rate less than 15 percent due to cancer relapse and metastasis [4-6]. Thus, identification of novel prognostic biomarkers and therapeutic targets is urgent.

FABP3 and FABP4 belong to fatty acid binding protein (FABP) family, which control the metabolism and transportation of long-chain fatty acids. FABP3 plays various roles in fatty acid transport, cell signaling, cell growth, and gene transcription [7]. Recent clinical studies have indicated the participation of FABP3 in tumor progression with conflicting functions. In breast cancer, FABP3 acts as a tumor suppressor gene: its expression was downregulated in breast cancer samples; FABP3 cDNA transfection in breast cancer cell lines leads to a modest anti-proliferative activity [8]; similarly, over-expression of FABP3 in embryonic cancer cells inhibits cell growth and leads apoptosis [9]. On the other hand, elevated FABP3 expression is associated with tumor progression, aggressiveness and poor prognosis in gastric carcinoma [10]. Thus far, there has been no study of FABP3 in NSCLC development and progression.

FABP4 has been linked to the development of metabolic syndrome, diabetes, and arteriosclerosis [11]. FABP4 is a prognostic predictor in infiltrating or invasive bladder cancer [12], and FABP4 is a potential therapeutic target for metastatic prostate cancers [13]. However, FABP4 in NSCLC has not been investigated.

Little is known about the expression of FABP3 and FABP4 in NSCLC. Here, we investigated the expression of these two markers and assessed their correlation with the clinicopathological features as well as prognosis in NSCLC. Finally, we also investigated the prognostic value of concurrent expression of FABP3 and FABP4 in NSCLC.

## RESULTS

### FABP3 and FABP4 mRNA expression was upregulated in NSCLC cancerous tissues

**Figure 1:**
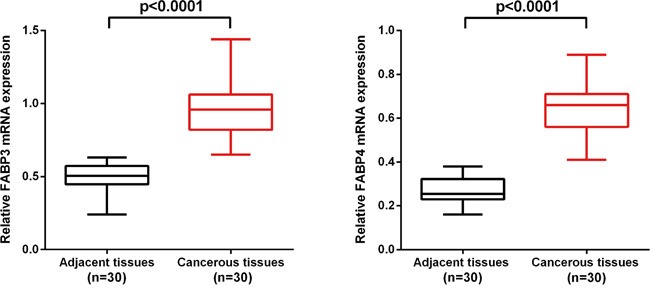
FABP3 and FABP4 mRNA level was significantly higher in NSCLC cancerous tissues than in matched adjacent non-cancerous tissues FABP3 and FABP4 mRNA was determined by qRT-PCR and relative quantification analysis by normalizing to GAPDH mRNA.

Quantitative reverse transcriptase polymerase chain reaction (qRT-PCR) was used to determine the relative expression of FABP3 and FABP4 mRNA in NSCLC cancerous (n=30) and matched adjacent non-cancerous tissues (n=30) by normalizing to the housekeeping gene GAPDH. FABP3 mRNA expression level was significantly higher in NSCLC cancerous tissues (0.64±0.12) than in matched non-cancerous tissues (0.27±0.06) (P<0.001). Similarly, FABP4 mRNA expression level was significantly higher in NSCLC cancerous tissues (0.97±0.18) than in matched non-cancerous tissues (0.50±0.09) (P<0.001) (Figure 1).

### FABP3 and FABP4 protein expression was significantly higher in NSCLC cancerous tissues

To verify our results on FABP3 and FABP4 mRNA expression, we examined FABP3 and FABP4 protein expression on 281 NSCLC cancerous tissues and 121 matched adjacent non-cancerous tissues by TMA-IHC. High FABP3 protein expression was detected in 59.43% (167/281) of NSCLC cancerous tissues, significantly higher than 9.92% (12/121) detected in adjacent noncancerous tissues (χ^2^=83.974, P<0.001). Similarly, high FABP4 expression was detected in 60.14% (169/281) of NSCLC cancerous tissue samples significantly higher than 8.26% (10/121) detected in adjacent non-cancerous tissues (χ^2^=92.156, P<0.001).

**Figure 2:**
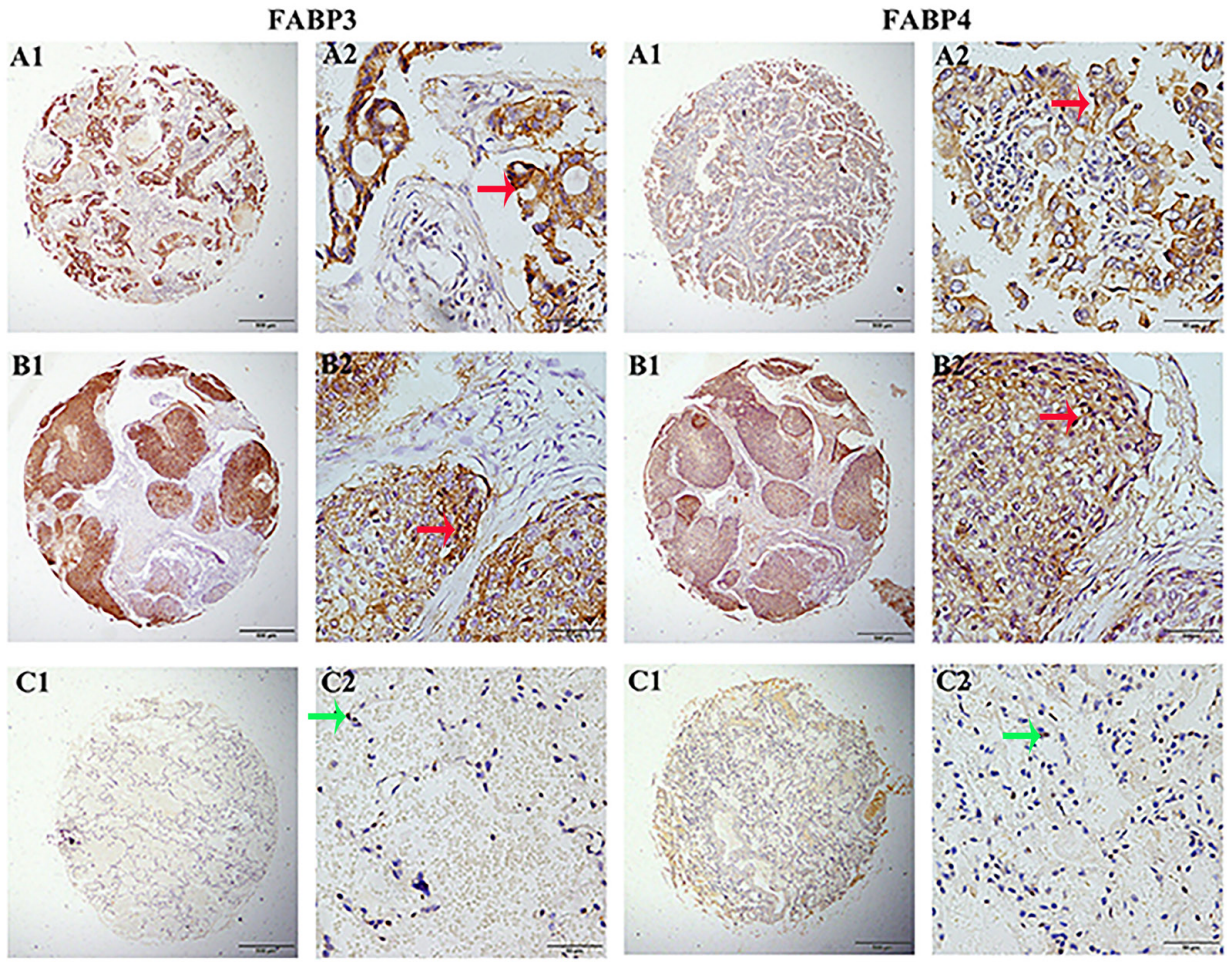
FABP3 and FABP4 protein detection in NSCLC tissues FABP3 and FABP4 protein was determined by TMA-IHC, **A1-A2.** adenocarcinoma tissues, strong positive for FABP3 and FABP4 protein expression; **B1-B2.** squamous cell carcinoma tissues, positive for FABP3 and FABP4 protein expression; **C1-C2.** matched adjacent non-cancerous tissue samples, negative for FABP3 and FABP4 protein expression. A1, B1, and C1 were ×40 magnification (bar=500 μm), A2, B2, and C2 were ×400 magnification (bar=50 μm). Red arrows indicate positive FABP3 or FABP4 protein expression on cancerous cell cytoplasm, and green arrows indicate negative FABP3 or FABP4 protein expression on adjacent non-cancerous cells.

Interestingly, we also observed differential expression patterns for FABP3 and FABP4 in cancerous and noncancerous tissues (Figure 2). In NSCLC cancerous tissues, FABP3 protein expression was mainly localized in the cytoplasm, and rarely detected in the nucleus. On the contrary, the majority of FABP3 protein expression was detected in the nucleus in adjacent non-cancerous tissues. Similar protein expression pattern was observed for FABP4.

### Relationships of FABP3 and FABP4 protein expression with clinicopathological characteristics of NSCLC patients

In order to elucidate the function of FABP3 and FABP4 protein expression during NSCLC progression, we correlated FABP3 and FABP4 protein expression with clinicopathological characteristics in NSCLC patients, including gender, age, histological type, TNM staging, smoking history, and differentiation. High FABP3 protein expression (FABP3+) (P=0.019), high FABP4 protein expression (FABP4+) (P=0.038), and high concurrent FABP3 and FABP4 protein expression (FABP3+/FABP4+) (P=0.035) were all significantly associated with higher TNM stage (Table 1). We also observed positive correlation between FABP3 and FABP4 protein expression in NSCLC cancerous tissues (r=0.364, P<0.001).

**Table 1:** Relationship between FABP3 and FABP4 protein expression in cancerous tissues and clinicopathological characteristics in NSCLC

Groups	n	FABP3 expression			FABP4 expression			FABP3+/FABP4+ expression		
		High(%)	Pearson χ^2^	p	High(%)	Pearson χ^2^	p	FABP3+/FABP4+(%)	Pearson χ^2^	p
Total	281	167(59.43)			169(60.14)			125(44.48)		
Gender			1.427	0.232		3.330	0.068		0.045	0.832
Female	85	46(54.12)			58(68.24)			37(43.53)		
Male	196	121(61.73)			111(56.63)			88(44.90)		
Age			0.349	0.555		0.212	0.645		0.053	0.819
≤ 60	110	63(57.27)			68(21.81)			48(43.64)		
> 60	171	104(60.82)			101(59.06)			77(45.03)		
Histological type			0.502	0.778		4.353	0.113		0.779	0.677
Adenocarcinoma	128	75(58.59)			86(67.19)			61(47.66)		
Squamous cell carcinoma	99	62(62.63)			54(54.55)			42(42.42)		
Others^a^	54	30(57.69)			29(55.77)			22(42.31)		
T			5.581	0.061		3.484	0.175		4.759	0.093
T1	119	62(52.10)			64(53.78)			45(37.81)		
T2	142	90(63.38)			92(64.79)			68(47.89)		
T3+T4	20	15(75.00)			13(65.00)			12(60.00)		
N			3.914	0.141		5.611	0.060		5.359	0.069
N0	163	89(54.60)			89(54.60)			63(38.65)		
N1	68	46(67.65)			44(64.71)			36(52.94)		
N2	50	32(64.00)			36(72.00)			26(52.00)		
TNM			7.921	0.019*		6.555	0.038*		6.728	0.035*
0-I	127	64(50.39)			69(54.33)			46(36.22)		
II	93	63(67.74)			55(59.14)			46(49.46)		
III-IV	61	40(65.57)			45(73.77)			33(54.10)		
Smoking			0.088	0.767		2.224	0.136		0.254	0.614
No	173	104(60.12)			110(63.58)			79(45.66)		
Yes	108	63(58.33)			59(54.63)			46(42.59)		
Differentiation			0.035	0.851		<0.001	0.983		0.739	0.390
Low	83	56(67.47)			50(60.24)			38(45.78)		
Middle and high	198	111(56.06)			119(60.10)			87(43.94)		

* P<0.05

a others, Adenosquamous carcinoma

### Elevated protein expression of FABP3 and FABP4 was correlated with poor prognosis in NSCLC

**Figure 3:**
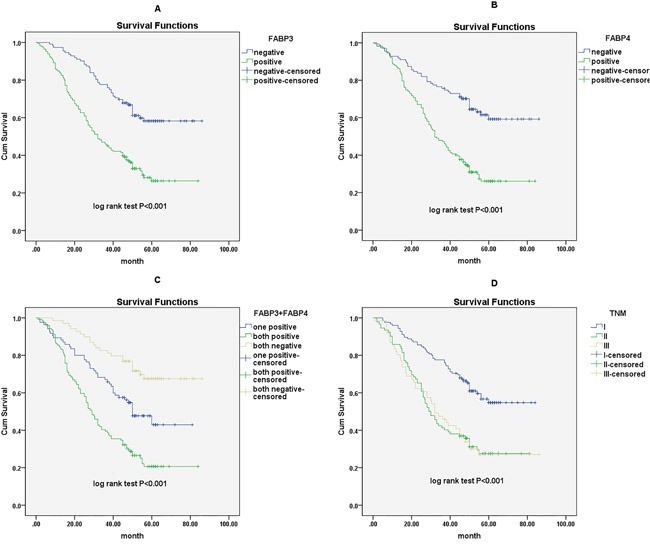
Survival curves of NSCLC patients by the Kaplan–Meier method and the log-rank test **A.** FABP3+ NSCLC patients (green line) had significantly worse overall survival than FABP3- patients (blue line); **B.** FABP4+ NSCLC patients (green line) had significantly worse overall survival than FABP4- patients (blue line); **C.** FABP3+/FABP4+ NSCLC patients (green line) had significantly worse overall survival than FABP3+ or FABP4+ patients (blue line) and FABP3-/FABP4- NSCLC patients (yellow line); **D.** NSCLC patients with advanced TNM stage (green line for stage III and yellow line for stage II) had significantly worse overall survival than patients with TNM stage I (blue line).

Finally, we used the Cox proportional hazards regression analysis to investigate the association between the FABP3 or FABP4 protein expression and prognosis in NSCLC patients. Of 281 NSCLC patients, 125 patients had concurrent high FABP3 and FABP4 protein expression in tumor (FABP3+/FABP4+), 86 patients had either high FABP3 or high FABP4 protein expression in tumor (FABP3+/FABP4- or FABP3-/FABP4+), and 70 patients had no high protein expression of FABP3 and FABP4 (FABP3-/FABP4-). Univariate analysis revealed that high FABP3 expression (HR, 2.544, 95% CI: 1.794-3.609; P<0.001), high FABP4 expression (HR, 2.634, 95% CI: 1.840-3.769; P<0.001), concurrent high FABP3 and FABP4 expression (HR, 2.032, 95% CI: 1.634-2.526; P<0.001), tumor status (T) (HR, 1.549, 95% CI: 1.206-1.991; P=0.001), lymph node metastasis (N) (HR, 1.526, 95% CI: 1.265-1.842; P<0.001), and TNM stage (HR, 1.576, 95% CI: 1.305-1.902; P<0.001) were all associated with poor five-year survival in NSCLC patients. High FABP3 expression (HR, 1.900, 95% CI: 1.316-2.744; P=0.001), high FABP4 expression (HR, 1.978, 95% CI: 1.353-2.893; P<0.001) and TNM stage (HR, 1.467, 95% CI: 1.213-1.774; P<0.001) remained significantly associated with poor five-year survival in multivariate analysis (Table 2). Kaplan-Meier survival curves further confirmed that NSCLC patients with high FABP3 protein expression, high FABP4 protein expression, or advanced TNM stage had significantly shorter overall survival time in NSCLC (Figure 3). Interestingly, NSCLC patients with concurrent high FABP3 and FABP4 protein expression (FABP3+/FABP4+) had poorer prognosis, and showed shorter survival time significantly than patients with either high FABP3 or high FABP4 protein expression (Figure 3).

**Table 2:** Univariate and multivariate analysis of different prognostic factors for 5-year survival in NSCLC patients

Varible	Univariate analysis			Multivariate analysis		
	HR	P	95%CI	HR	P	95%CI
FABP3 expression	2.544	<0.001*	1.794-3.609	1.900	0.001*	1.316-2.744
High vs low						
FABP4 expression	2.634	<0.001*	1.840-3.769	1.978	<0.001*	1.353-2.893
High vs low						
FABP3/FABP4 expression	2.032	<0.001*	1.634-2.526	0.978	0.867	0.758-1.262
Both positive vs one positive vs both negative						
Sex	1.169	0.376	0.827-1.651			
Female vs male						
Age	1.048	0.774	0.761-1.443			
≤ 60 vs > 60						
Histological type	1.155	0.176	0.937-1.422			
Ad vs Sq vs others						
T	1.549	0.001*	1.206-1.991			
T1 vs T2 vsT3+T4						
N	1.526	<0.001*	1.265-1.842			
N0 vs N1 vs N2						
TNM	1.576	<0.001*	1.305-1.902	1.467	<0.001*	1.213-1.774
I vs II vs III-IV						
Smoking history	0.801	0.183	0.578-1.110			
Yes vs No						
Differentiation	1.025	0.883	0.737-1.427			
Well and moderate vs poor						

* P<0.05. TNM stage contains T stage and N stage, therefore, they were not included in the multivariate analysis.

Ad: Adenocarcinoma; Sq: Squamous cell carcinoma.

## DISCUSSION

In the present study, we found that both mRNA and protein expression of FABP3 and FABP4 were significantly higher in NSCLC cancerous tissues than in adjacent non-cancerous tissues. High protein expression of FABP3 and FABP4 in NSCLC as well as concurrent high protein expression of FABP3 and FABP4 was correlated with higher TNM stage and associated with shorter overall survival. Our results indicated that high FABP3 and FABP4 protein expression were independent poor prognostic factors of NSCLC. Finally, we showed positive correlation between FABP3 and FABP4 protein expression.

The principal role of FABPs is to regulate fatty acid uptaking and intracellular trafficking. Each member of FABP family is a tissue-specific intracellular fatty acid carrier protein. FABPs regulate the concentration of intracellular free fatty acids and maintain a stable internal environment for lipid metabolism [14]. Recent studies have indicated that fatty acid metabolism is involved in tumorigenesis and tumor progression of many types of cancer [15-17], by influencing tumor proliferation and tumor migration. FABPs are differentially expressed in various cancers and are suggested potential tumor biomarkers [10, 18-22].

Besides its functions in lipid transport, storage, and metabolism [23], FABP3 is also involved in cell growth and proliferation. FABP3 expression is elevated after myocardial injury, and is widely used as a marker of acute myocardial infarction and stroke [24-26]. More recent studies demonstrated the interaction between FABP3 and cancer biology in various types of cancer, including breast cancer [8], gastric carcinoma [10], brain tumor [27], and small cell lung cancer [28]. Besides binding to long-chain fatty acids, FABP4 is also capable of binding to a variety of hydrophobic compounds, including cycloxygenases and oxidative products of fatty acids. It functions in both glucose and lipid metabolism [29, 30], and is involved in signal transduction [31], as well as in cell proliferation and apoptotic processes [32]. Its involvement in tumorigenesis has been investigated in bladder cancer [12], breast cancer [33, 34], ovarian cancer [35, 36], and oral squamous cell cancer [37]. However, there have been few studies of FABPs in NSCLC patients. Our results suggest that FABP3 and FABP4 could be promising biomarkers in NSCLC.

Several studies have demonstrated the association between FABP4 expression and poor prognosis in several tumors [38-40], which were consistent with our current findings. However, the role of FABP3 in tumor is controversial: FABP3 can act as either a tumor suppressor or an oncogene depending on tumor types. In breast cancer and embryonic cancer cells, FABP3 acts as a tumor suppressor [41]: FABP3 inactivation inhibits apoptosis and promotes proliferation; while FABP3 acts as an oncogene in gastric carcinoma, brain tumor and small cell lung cancer [42]. Our study indicated that FABP3 acts as an oncogene in NSCLC.

The underlying molecular mechanism of FABP3 and FABP4 in tumorigenesis has been investigated in a few studies. Song GX et al reported that in embryonic cancer cell, FABP3 promoted apoptosis through inducing mitochondrial impairment [7]. Hu J et al described that in ovarian cancer, FABP4 promoted fatty acids absorption from adipocytes for rapid tumor growth in adipocyte-rich microenvironment [43]. Further in vitro functional studies are needed to elucidate the molecular mechanisms of FABP3 and FABP4 in NSCLC. Our finding of the positive correlation between FABP3 and FABP4 expression suggest that these two proteins may act in the same signaling pathway in promoting tumor progression.

Our study confirms that fatty acid metabolism pathway is involved in tumorigenesis again. It is well documented that tumor cells have unique metabolic requirement comparing to normal cells [44]. Earlier studies have focused on glucose and glutamine metabolism pathways during tumor development. Latest research has suggested that fatty acid metabolism also plays an important role in various aspects of tumor cell proliferation, transformation and migration [45]. Mechanistically, the high proliferation rate of tumor cells requires a large amount of fatty acids [46], not only as energy source, but also as building blocks for large biomolecules [47, 48]; many lipid molecules can act as signaling molecules that induce carcinogenesis [49]. FABPs, a super-family of lipid binding protein, have important functions on the uptake, transport and oxidation of fatty acid and their derivatives. Therefore, FABPs represent potential novel targets as cancer biomarkers and therapeutic targets. Future studies are needed to elucidate the mechanism of FABP3 and FABP4 in fatty acid metabolism and in NSCLC tumorigenesis.

Our study has several limitations. First, our study is a retrospective correlation study, thus we do not know whether FABP3 and FABP4 protein expression is the driving force of NSCLC progression or the consequence of NSCLC development. Larger prospective studies are needed to distinguish these two scenarios. Second, protein quantification by IHC analysis is subjective and semi-quantitative; our results on FABP3 and FABP4 protein expression should be independently validated by other methods. Finally, we did not perform in vitro functional studies to elucidate the molecular mechanisms of FABP3 and FABP4 expression and NSCLC development.

In conclusion, our results demonstrated that both high FABP3 and FABP4 protein expression is associated with poor prognosis in NSCLC. Our study provides rationale to further investigate the involvement of fatty acid metabolism signaling pathway in NSCLC development as well as FABP3 and FABP4 as potential prognostic markers and therapeutic targets in NSCLC.

## MATERIALS AND METHODS

### Tissue samples

NSCLC cancerous tissues (n=281) and matched adjacent non-cancerous tissues (n=121) were collected as archived formalin fixed, paraffin embedded samples from human clinical biobank in Affiliated Hospital of Nantong University, Jiangsu Province, China. The ages of patients ranged from 35 to 83 years old, with a median of 62.1 years old, including 85 women and 196 men. All cases were histologically diagnosed with NSCLC. None of the patients had received neo-adjuvant chemotherapy, radiotherapy or immunotherapy before surgery. All patients had signed informed consent before surgery and the Research Ethics Committee approved this study.

### Quantitative real-time reverse transcription PCR (qRT-PCR)

The Department of Pathology at the Affiliated Hospital of Nantong University provided the fresh frozen NSCLC cancerous tissues (n=30) and matched adjacent non-cancerous tissues (n=30). Total RNA was extracted from these samples using Trizol reagent (Invitrogen, Carlsbad, CA, USA), and about 2 μg of total RNA was reverse transcribed into cDNA using High-Capacity cDNA Reverse Transcription kit (Applied Biosystems). The following oligonucleotide primers were used for PCR amplification: FABP3 forward: 5′-CAC CTG GAA GC TAG TGG ACA-3′, FABP3 reverse: 5′-TTC CCT CCA TCC AGT GTC AC-3′. FABP4 forward: 5′-CTG GTG GTG GAA TGC GTC ATG A-3′, FABP4 reverse: 5′-CAA CGT CCC TTG GCT TAT GCT CTC T-3′. GAPDH forward: 5′-GGT AGA CAA GTT TCC CTT-3′, GAPDH reverse: 5′-ATA TGT TCT GGA TGA TTC T-3′.

### Tissue microarrays (TMA) construction and immunohistochemistry (IHC) analysis

NSCLC cancerous and matched adjacent non-cancerous tissues were prepared for TMAs. The manual Tissue Microarray System Quick-Ray (UT06, UNITMA, Korea) was used to generate TMAs. Core tissue samples (diameter of 2 mm) were obtained from paraffin embedded tissue sections and deposited in paraffin-recipient blocks. Before immunohistochemical processing, the tissue microarray specimens were cut into 4-μm-thick sections and placed on the super frost charged glass microscope slides. IHC was carried out as described before [50]. Briefly, the slides were incubated with the primary antibodies for FABP3 (1:100; Abcam, Cambridge, MA, USA) and FABP4 (1:100; Abcam, Cambridge, MA, USA) overnight at 4°C. Subsequently, the slides were incubated with anti-mouse IgG secondary antibody (Abcam) for FABP3 and horseradish-peroxidase-conjugated rabbit IgG (Abcam) for FABP4. Diaminobenzidine solution was used for the color development.

### TMA-IHC data evaluation

IHC staining was scored independently by two individuals, who were blinded to each other's findings and the clinical characteristics of these samples. Protein expression was analyzed by the staining intensity and the percentage of cells stained. The staining intensity was scored as 0 (no), 1 (weak), 2 (moderate), and 3 (strong), then multiplied by the percentage of positive cells. Therefore, the final score ranged from 0 (no cells with any staining) to 300 (all cells with strong staining). The protein expression was then dichotomized into low protein expression and high protein expression using a cutoff point determined by the X-file software (Rimm Laboratory at Yale University; http://www.tissuearray.org/rimmlab) as previously introduced [51]. In this study, the cutoff points for FABP3 and FABP4 were 90 and 120 respectively: expression score equal or higher than the cutoff point was considered high expression, while expression score lower than the cutoff point was considered low expression.

### Statistical analysis

Pearson χ^2^ test was used to evaluate the correlation of FABP3 and FABP4 expression with clinicopathological characteristics of NSCLC patients. Spearman correlation test was performed to determine the correlation between FABP3 and FABP4 protein expression. Both univariate and multivariate Cox regression analysis were used to identify independent prognostic factors. Kaplan–Meier and the log-rank tests were used to calculate survival curves. P value of less than 0.05 was regarded as statistically significant. All statistical analyses were accomplished in SPSS version 20.0 statistical software (SPSS Inc., Chicago, IL, USA).
